# Multi-Omics Perspectives on Testicular Aging: Unraveling Germline Dysregulation, Niche Dysfunction, and Epigenetic Remodeling

**DOI:** 10.3390/cells14120899

**Published:** 2025-06-13

**Authors:** Aris Kaltsas

**Affiliations:** Third Department of Urology, Attikon University Hospital, School of Medicine, National and Kapodistrian University of Athens, 12462 Athens, Greece; ares-kaltsas@hotmail.com

**Keywords:** testicular aging, spermatogenesis, Sertoli cells, Leydig cells, oxidative stress, male fertility decline

## Abstract

Male reproductive aging proceeds gradually and involves complex alterations across germ cells, somatic cells, and the testicular niche. Multi-omics analyses highlight shifts in spermatogonial stem cell dynamics, diminished sperm quantity and quality, and reconfigured support from Sertoli and Leydig cells. These somatic cells show numerical declines and exhibit senescence-associated changes that amplify inflammatory signals and compromise blood–testis barrier integrity. Concurrently, fibrosis and heightened immune cell infiltration disrupt intercellular communication, contributing to further deterioration of spermatogenesis. Epigenetic remodeling—including DNA methylation drift, histone modification imbalances, and altered small non-coding RNA profiles—adds another dimension, reducing sperm integrity and potentially exerting transgenerational effects on offspring health. Observed hormonal changes, such as reduced testosterone and INSL3 production by aging Leydig cells, reflect the additional weakening of testicular function. These multifactorial processes collectively underlie the drop in male fertility and the increased incidence of adverse outcomes, such as miscarriages and developmental anomalies in the offspring of older fathers. Research into mitigation strategies, including interventions targeting senescent cells, oxidative stress, and inflammatory pathways, may slow or reverse key mechanisms of testicular aging. These findings underscore the importance of understanding the molecular hallmarks of male reproductive aging for preserving fertility and safeguarding offspring well-being.

## 1. Introduction

Male reproductive aging is a gradual process marked by declines in fertility, hormonal output, and sperm quality—unlike the abrupt cessation of ovarian function and menstruation seen in female menopause [1]. This prolonged timeframe allows sperm production to continue throughout the lifespan, but reduced fertility, diminished androgen levels, and compromised sperm quality frequently emerge in later years [2]. Such changes are not confined to germ cells; they also reflect substantial shifts in the testicular milieu, encompassing Sertoli cells, Leydig cells, peritubular myoid cells, immune populations, and the extracellular matrix [3,4]. These components function as a unified system, the balance of which is essential for maintaining spermatogenesis and hormonal outputs.

Testicular aging is initiated at characteristic ages across diverse species, reflecting conserved biological hallmarks. In humans, subtle declines in testicular function—including reduced Leydig cell activity, declining testosterone levels, and modest decreases in sperm production—become evident around the fifth decade of life, intensifying thereafter with histological and hormonal changes into older age [5,6]. In rodent models, this aging process is compressed: mice display initial testicular aging features by approximately 12 months of age, characterized by stem cell attrition, decreased spermatogenesis, and structural remodeling [7]. Similarly, rats begin exhibiting pronounced fertility declines and hormonal disruptions between 15 and 18 months [8]. Non-human primates, such as rhesus macaques, parallel human reproductive aging patterns, demonstrating measurable declines in testicular function, including lower testosterone and reduced fertility, typically emerging around 15–20 years of age [9,10].

**Figure 1:**
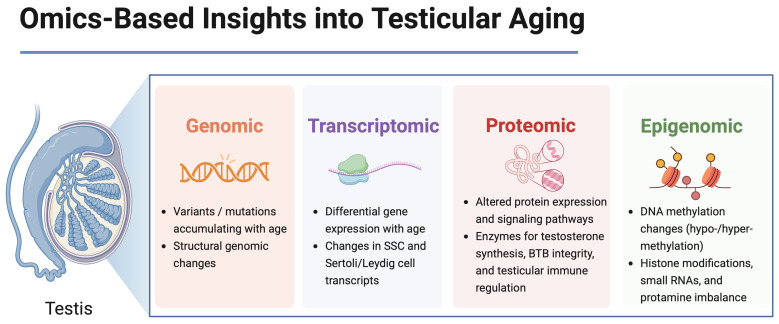
Multi-omics approaches reveal molecular layers of testicular aging. The testis consists of interstitial Leydig cells responsible for androgen production and seminiferous tubules containing Sertoli cells, spermatogonial stem cells (SSCs), and differentiating spermatogenic cells. Multi-omics analyses (genomic, transcriptomic, proteomic, and epigenomic) focus primarily on SSCs, Sertoli cells, and Leydig cells due to their critical roles in spermatogenesis and hormonal regulation. SSC: spermatogonial stem cell, essential for ongoing sperm production and renewal of the germ cell line. BTB: blood–testis barrier, a specialized junction formed primarily by Sertoli cells, which protects developing germ cells by maintaining a controlled microenvironment and preventing immunological interference. Created in BioRender. Kaltsas et al., 2025. Available online: https://BioRender.com/pgc5vvd (accessed on 31 May 2025).

Age-related transformations in the germline have been studied extensively, and recent advances in high-throughput methods have offered more nuanced insights. Transcriptomics, proteomics, and epigenomic analyses collectively suggest that aging induces the widespread remodeling of gene expression and protein profiles within germ cells [11]. These omics layers—each addressing distinct molecular dimensions—are visualized in Figure 1 to illustrate their complementary contributions to testicular aging.

Critical pathways governing cell cycle progression, DNA repair, and metabolic homeostasis are notably affected, with an associated escalation of stress and inflammatory signals [12]. These disruptions become increasingly pronounced in older males, where a gradual decline in spermatogonial stem cells leads to fewer mature sperm and reduced functional competence.

The testicular microenvironment is also affected by age-related disruptions that worsen fertility outcomes. Sertoli cells experience both quantitative and qualitative decline, as evidenced by fewer cells per seminiferous tubule and the compromised structural integrity of the blood–testis barrier [13]. Studies indicate that these cells display altered transcriptional activity, heightened markers of senescence, and a compromised capacity to nurture germ cells. Leydig cells likewise undergo functional depletion, ultimately contributing to declining testosterone and other key hormones necessary for spermatogenic support. While remaining Leydig cells sometimes retain the ability to synthesize androgens, their overall efficiency deteriorates with age [14,15].

Parallel to these cellular developments, the testicular niche undergoes progressive fibrosis, changes in basement membrane thickness, and increased immune cell infiltration [16]. Signaling cascades that coordinate spermatogenic events—such as those involving Activin, Hedgehog ligands, and c-Kit—are downregulated, while pro-inflammatory and senescence-associated secretory factors are elevated in older testes. These intercellular disruptions create an unfavorable environment for germ cell proliferation and differentiation [17].

Further molecular insights come from recent work demonstrating that deficiency in retinoid acid receptor-related orphan receptor alpha (RORα) accelerates testicular aging, characterized by the morphological deterioration of seminiferous tubules, germ cell apoptosis, Leydig cell dysfunction, and increased fibrosis. Interestingly, melatonin supplementation markedly mitigates these aging-associated alterations via anti-inflammatory, antioxidant, and anti-apoptotic mechanisms [18].

Epigenetic and small RNA factors are also implicated in the decline in male reproductive potential. Age-specific shifts in sperm DNA methylation patterns and histone modifications can undermine fertilization outcomes and embryonic development. Alterations in microRNAs and PIWI-interacting RNAs further influence transcriptional regulation, with possible transgenerational effects. Importantly, oxidative stress has emerged as a potent upstream driver of such epigenetic dysregulations, wherein excessive reactive oxygen species (ROS) disrupt DNA methylation, histone marks, and small RNA biogenesis, ultimately impairing spermatogenesis and male fertility [19]. Some investigations reveal that the offspring of older fathers exhibit a greater incidence of developmental abnormalities, potentially linked to these epigenetic disruptions [3].

Collectively, these findings underscore the intricate interplay between germ cells and their supportive niche in the aging testis. The synthesis of multi-omics data from diverse models has revealed that a network of molecular, cellular, and epigenetic factors underpins the gradual decline in male fertility. The insights derived from this research provide a basis for identifying biomarkers of testicular aging and guiding strategies aimed at mitigating its impact on reproductive health and offspring well-being.

## 2. Age-Related Gene Expression Changes in Germ Cells

Bulk transcriptome analyses have documented broad shifts in testicular gene expression with age. For example, an RNA-seq study of mouse testes (ages 3, 6, 12, and 18 months) identified over 1500 mRNAs and ~700 long non-coding RNAs (lncRNAs) with differential expression during aging [20]. Many transcripts showed gradual, continuous changes in expression as the mice aged, rather than abrupt switches [20]. Notably, genes related to spermatogenesis were enriched among those increasing or decreasing with age [20]. This indicates that the germline transcriptome undergoes a steady remodeling as animals transition from young adulthood to middle age. In rats, aging was also shown to alter spermatid gene expression and perturb spermatogenic processes [21]. Taken together, bulk sequencing suggests that aging testes maintain overall transcriptional activity but with subtle shifts in the balance of germ cell gene programs (e.g., regulators of meiosis, spermiogenesis, and gamete function).

Single-cell transcriptomic studies provide more refined insight into how specific germ cell populations are affected by age. Dong et al., 2022, performed the first single-cell RNA sequencing (scRNA-seq) comparison of human testes from young vs. older men [22]. They found that aging has an inconsistent impact on spermatogenic cells: Some older men retained full spermatogenesis, whereas others showed obvious impairment [22]. In those older individuals with reduced sperm production, a significant decline in the number of spermatogonial stem cells (SSCs) was observed, implicating SSC loss in age-related spermatogenic failure [22]. Similarly, a single-nucleus transcriptomic atlas of primate (macaque) testes revealed a marked attrition of the SSC reservoir in aged males, along with disrupted gene expression programs for meiosis and spermiogenesis [23]. In that study, Huang et al., 2023, showed that germ cells from old monkeys had molecular signatures of impaired differentiation at multiple stages, consistent with fewer stem cells and incomplete germ cell development in older testes [23]. These single-cell data support a model in which male germline stem cells gradually decline in number and function with age, leading to compromised maintenance of spermatogenesis.

In addition to stem cell loss, aged germ cells that do complete spermatogenesis may exhibit altered gene expression impacting sperm function. For instance, genes involved in sperm capacitation, acrosomal function, and fertilization can be misregulated in sperm from older vs. younger males [20]. In one study, older mice showed an accumulation of spermatogonial clones that failed to produce sperm, occupying niches that would otherwise support functional spermatogenesis [24]. This clonal expansion of nonproductive germ cells in aged testes further diminishes the sperm output and genetic diversity of the sperm pool [24]. Overall, age-associated transcriptomic changes in germ cells include the downregulation of key differentiation factors, upregulation of stress and damage response genes, and selection for clones of stem cells with reduced spermatogenic capacity. These changes underpin the declining quantity and quality of sperm observed in older males. A comparative overview of primary omics studies investigating testicular aging across species is provided in Table 1.

## 3. Sertoli Cell Aging and Transcriptomic Alterations

Sertoli cells (SCs) are the somatic “nurse” cells within seminiferous tubules that physically and metabolically support developing germ cells. Aged Sertoli cells undergo both quantitative and qualitative changes that can adversely affect spermatogenesis. Morphometric analyses in humans show that the number of Sertoli cells significantly declines in older men [14]. In one study, testis biopsies from elderly men had far fewer Sertoli cells (per cross-section) compared to younger men, paralleling the loss of germ cells and the shrinkage of the tubule diameter with age [14]. This reduction in SC count means fewer support cells for each germ cell, potentially contributing to the drop in sperm production. Moreover, aged Sertoli cells exhibit morphological signs of dysfunction—including accumulated vacuoles, organelle aging, and the disorganization of the blood–testis barrier—as reported in histological studies [26].

Transcriptomic profiling indicates that Sertoli cells are among the most age-susceptible cell types in the testis. In the primate single-nucleus RNA-seq atlas, Sertoli cells showed the most dramatic age-related shift in gene expression among all testicular cell types [23]. Many genes essential for the support role of SCs were downregulated in old males. In particular, the transcription factor *WT1* (Wilms’ Tumor 1)—a master regulator of Sertoli cell identity and function—was markedly reduced in aged SCs [23]. The loss of WT1 expression was associated with accelerated cellular senescence in Sertoli cells, the disruption of tight junctions between SCs, and a breakdown of normal SC identity markers [23]. Since tight junctions between Sertoli cells form the blood–testis barrier (BTB) and create a specialized environment for germ cells, their disruption with age can expose germ cells to harmful immune or toxic factors. Indeed, older men’s seminiferous tubules often show focal BTB leaks and impaired barrier integrity [26]. The aged SC transcriptional profile also reflects metabolic alterations—Dong et al. noted that metabolic and nutrient signaling pathways in Sertoli cells are significantly altered in older males [22]. These changes could affect the energy substrates and growth factors that Sertoli cells provide to developing sperm, thus indirectly hampering spermatogenesis.

Another hallmark of Sertoli cell aging is a pro-inflammatory and senescent phenotype. Aged SCs presented the upregulated expression of many immune- and stress-related genes—such as cytokines and components of the senescence-associated secretory phenotype (SASP), a collection of pro-inflammatory factors secreted by senescent cells—that are not highly expressed in young supportive Sertoli cells [22,26]. For example, SASP factors have been identified as differentially expressed in older human testes, with Sertoli cells likely contributing to this pro-inflammatory secretome [26]. A recent analysis of human testis single-cell data identified insulin-like growth factor binding protein 7 (IGFBP7) as an SASP factor strongly upregulated in aging testes that is mainly expressed by Leydig and other interstitial cells, but its elevation reflects a generally inflammatory milieu [26]. Chronic low-grade inflammation within the aging testis can create a less appropriate environment for germ cells. Furthermore, age-related declines in Sertoli cell-secreted factors that promote germ cell development have been reported. For instance, Sertoli cells produce c-Kit ligand (SCF) and glial cell line-derived neurotrophic factor (GDNF) to support spermatogonial stem cells; studies suggest these pro-spermatogenic signals diminish with age, leading to weaker maintenance of the stem cell pool [29]. In summary, aging Sertoli cells are fewer in number and exhibit altered gene expression characterized by the loss of supportive functions and gain of senescent/inflammatory traits, which together compromise the nurturing microenvironment required for continuous sperm production.

## 4. Leydig Cell Aging and Steroidogenic Decline

Leydig cells (LCs) in the testis are responsible for testosterone production and also produce other factors such as INSL3 that influence reproductive physiology. Aging has a well-documented effect on Leydig cell function, as men experience a decline in testosterone levels, which is partly due to changes within the testes. Stereological analyses indicate that the number of Leydig cells significantly decreases in aged men, in parallel with Sertoli cell loss [14]. A 2020 study found aged testes contained significantly fewer Leydig cells per testis, along with a corresponding reduction in *INSL3* expression—an insulin-like peptide hormone produced by LCs—proportional to the extent of cell loss [14]. Lower INSL3 in blood is a biomarker of Leydig cell function, and its decline with age reflects diminished Leydig cell population and maybe function.

Notably, residual Leydig cells from older men often retain near-normal testosterone production capacity when tested in vitro [14]. This suggests that the age-related drop in testosterone in vivo is largely due to fewer Leydig cells and possibly altered regulation, rather than an inability of each cell to synthesize the hormone [14]. Nevertheless, transcriptomic studies do show molecular changes in aged LCs that could impair their steroidogenic output or broader role. Dong et al. observed a reduction in Leydig cells and testosterone biosynthesis in older human males, as indicated by scRNA-seq profiles and clinical data [22]. They specifically noted the downregulation of key components of the Hedgehog signaling pathway in aged human Leydig cells [22]. Desert Hedgehog (DHH) from Sertoli cells is important for fetal Leydig cell development and adult LC maintenance; reduced DHH signaling in aged LCs may contribute to their functional decline [22]. Along with that, aged LCs showed signs of senescence and increased expression of inflammatory genes [22]. The SASP factor IGFBP7, for instance, was identified in one study as prominently expressed by aged Leydig cells and accumulating in the interstitial compartment of old testes [26]. Excess IGFBP7 could potentially modulate local IGF/insulin signaling and impair steroidogenesis or cell survival through autocrine mechanisms, although its specific effects remain to be fully elucidated.

At the proteomic level, aging seems to affect enzymes and pathways in Leydig cells that are crucial for testosterone synthesis. Some studies implicate increased oxidative stress and mitochondrial dysfunction in aged LCs [36]. For example, the age-related impairment of redox homeostasis has been noted in old rodent Leydig cells, with antioxidants shown to partially restore steroidogenic capacity in experimental models [37]. Moreover, certain cell signaling pathways such as p38 mitogen-activated protein kinase (p38 MAPK) become overactive in aging LCs and contribute to reduced steroidogenesis and accelerated cell senescence [35]. Blocking such pathways has been shown in animal studies to partially restore testosterone production in aging testes [35].

Leydig cell aging also has endocrine repercussions beyond testosterone. The decrease in INSL3 with age is linked to conditions such as late-onset hypogonadism, and lower INSL3 could affect bone and muscle homeostasis in older men [14]. In summary, aging Leydig cells decrease in number and display altered gene expression, characterized by reduced steroidogenic signaling and elevated stress and inflammatory markers. While the remaining cells might maintain some steroidogenic potential, the overall hormonal output (testosterone, INSL3) of the testis declines. These changes in the Leydig cell population underlie the hypogonadism and fertility reduction seen in older males.

## 5. Changes in the Testicular Niche and Intercellular Signaling with Age

The testicular microenvironment—including peritubular myoid cells, blood vessels, immune cells, and the basement membrane (BM) architecture—undergoes significant modifications with aging that can indirectly influence both germ and somatic cell function. One striking early change occurs in the peritubular myoid cells and tubular wall. A single-cell analysis across the human lifespan noted an initial “wave” of aging changes appearing in men in their 30s, characterized by the thickening of the tubular basement membrane and altered gene expression in peritubular cells [25]. This thickening of the BM around seminiferous tubules—a hallmark of testicular aging—may hinder the exchange of nutrients and signaling molecules between the interstitial vasculature and the seminiferous epithelium [25]. By the 50s, a second wave of changes was evident: Somatic cells in the interstitial niche exhibited functional declines, such as dysregulated steroid metabolism in Leydig cells and heightened immune/inflammatory responses in testicular macrophages [25]. These intercellular changes suggest that communication networks within the aging testis become perturbed.

Cell–cell signaling pathways that are critical for coordinating spermatogenesis show measurable alteration with age. Using scRNA-seq data and ligand–receptor interaction analysis, Nie et al. found that Activin and KIT ligand signaling between Sertoli cells and spermatogonia is significantly weakened in older human testes [29]. Activin, a factor belonging to the TGF-β family, and KITLG, the stem cell factor produced by niche cells, normally promote spermatogonial proliferation and differentiation. In aging, reduced signaling through these pathways likely contributes to impaired SSC maintenance and the diminished progression of spermatogonia into meiosis [29]. Similarly, as mentioned, diminished DHH signaling from Sertoli to Leydig cells was observed, which could exacerbate Leydig cell loss and androgen decline [25]. Another study in mice reported reduced Pleiotrophin (PTN) signaling in aged testes, which was associated with impaired spermatogenesis [38]. Pleiotrophin is a growth factor that may support spermatogonial or Sertoli cell function; its reduction with age could be another factor in germ cell attrition.

The immune environment of the testis—normally tightly regulated to prevent anti-sperm immune reactions—also shifts with age. In mammals, testicular macrophages in older males tend to adopt a more inflammatory phenotype, secreting cytokines that can affect Leydig and Sertoli cell function [25]. Indeed, human aging testes have been noted to contain elevated levels of pro-inflammatory cytokines in the somatic cells [25]. This inflammation may reflect a testicular SASP response, in which senescent cells—such as aged Sertoli or Leydig cells—secrete factors such as IL-6, IGFBP7, and others [26]. While some immune surveillance is necessary, chronic inflammation in the testicular niche could impair spermatogonial niches or even trigger fibrosis, further disrupting cell–cell communication.

Structural changes also occur. As mentioned, basement membrane thickening around tubules and reduced vascular perfusion might limit oxygen and nutrient delivery to the germinal epithelium in aged testes. Ultrastructural studies have noted the degeneration of the vasculature and increased deposition of collagen in the interstitium with age [25]. These changes can create a hypoxic or nutrient-poor microenvironment that stresses the germ cells.

In summary, the aging testicular niche experiences a breakdown in the finely tuned intercellular signaling network that supports fertility. Key supportive signals—such as Activin, GDNF, SCF, and Hedgehog—are diminished with age, while inhibitory or damaging signals, including inflammatory cytokines, ROS, and SASP factors, are elevated [22,25]. Physical changes such as fibrosis and barrier disruption further isolate germ cells from the support they need. Together, these changes culminate in a suboptimal microenvironment for spermatogenesis in aging males [23], even if some stem cells and somatic cells remain present. Addressing these niche-level changes through anti-fibrotic or anti-inflammatory interventions may be crucial for mitigating age-related male infertility in the future.

## 6. Epigenetic Changes in the Aging Male Germline

Beyond mRNA transcripts and proteins, small non-coding RNAs in sperm and testes are emerging as critical regulators of fertility and potential mediators of paternal age effects on offspring. MicroRNAs (miRNAs) and piwi-interacting RNAs (piRNAs) are highly abundant in germ cells and can influence gene expression and transposon activity, respectively. Growing evidence indicates that the profile of these small RNAs shifts with age in the male germline.

MicroRNAs in sperm: Several studies in the past 5–10 years have profiled sperm miRNAs from younger vs. older males. In humans, Paoli et al., 2019 reported distinct age-related changes in sperm: Older men showed lower levels of specific miRNAs, including miR-146a, miR-122, and others, compared to younger men [31]. These changes were accompanied by other age-related effects, including increased sperm DNA fragmentation and an altered protamine 1-to-protamine 2 ratio in the sperm of older men [39]. Since protamine packaging is essential for sperm chromatin integrity, this imbalance indicates epigenetic dysregulation in aging sperm. A more recent large study of 333 men also found age-dependent shifts in sperm miRNA content, and some of these miRNA changes correlated with declines in standard semen parameters [40]. Notably, altered small RNA profiles have also been identified in severe forms of male infertility, such as idiopathic azoospermia, further highlighting the potential of sperm-borne small RNAs as biomarkers of testicular dysfunction [41]. Thus, in humans there is evidence that advancing age subtly reprograms the small RNA payload of sperm.

In mice, Miyahara et al., 2024 conducted a comprehensive analysis of how paternal age alters sperm miRNA profiles [42]. They compared sperm from young (3-month-old), mid-age (12-month-old), and old (20-month-old) mice and identified dozens of miRNAs that changed significantly in abundance with age [42]. Some miRNAs increased in old sperm, while others decreased. Importantly, many of the affected miRNAs are known to regulate genes involved in neural development and are delivered to the zygote at fertilization [42]. For example, the study noted that miR-466j and miR-24-3p—which target the autism-associated genes *Oxtr* (oxytocin receptor) and *Gabrb2* (GABA receptor subunit), respectively—were dysregulated in old male sperm [42]. This finding provides a molecular link between older fathers and the higher incidence of neurodevelopmental disorders in their children (since changes in sperm miRNAs could impact early embryonic gene regulation) [42]. Overall, the mouse data reinforce that paternal aging induces broad shifts in sperm miRNA repertoires, potentially affecting not only the father’s fertility but also the developmental trajectories of offspring. These insights echo the emerging paradigm of paternal origins of health and disease (POHaD), analogous to maternal effects.

In addition to miRNAs, other small RNAs such as piRNAs—which are essential for transposon repression in the germline—may be affected by aging, although direct evidence remains limited. PiRNA biogenesis factors could decline in efficiency with age, leading to incomplete transposon silencing. In aged somatic tissues, transposable elements often become de-repressed; a similar phenomenon in aging germ cells could jeopardize genome integrity. Indirect evidence suggests that aging may impact piRNA biology; for instance, the treatment of adult mice with rapamycin—a compound known to mimic certain aspects of aging—has been shown to reduce the levels of piRNAs and piRNA pathway proteins in the testis [43]. While that was a pharmacological intervention, it suggests the possibility that an aging metabolic environment affects small RNA pathways. It is reasonable to hypothesize that older male germ cells accumulate slight defects in piRNA pathway function, potentially leading to increased transposon activity or genomic instability in sperm. This remains an area for future research, but it underscores that the epigenetic landscape of germ cells (small RNAs, DNA methylation, and chromatin state) is dynamic with age.

Indeed, beyond small RNAs, extensive epigenomic remodeling has been observed in the sperm and germ cells of aging males. DNA methylation patterns in sperm change markedly with paternal age. A 2023 genome-wide bisulfite sequencing study of human sperm identified 1565 differentially methylated regions (DMRs) associated with age, with the vast majority (74%) being hypomethylated in older men’s sperm and about 26% *hyper*methylated [27]. These age-DMRs were nonrandomly distributed—many occurred in gene regulatory regions, and the genes affected were enriched for developmental and neural functions [27]. Notably, although individual studies report varying sets of genes with age-dependent methylation, cross-study comparisons have identified several hundred genes that consistently exhibit age-associated methylation changes in sperm. These genes are implicated in key processes such as neurodevelopment, metabolism, and spermatogenesis [27]. Such changes in the sperm epigenome are thought to contribute to the increased risks of infertility, miscarriage, and certain diseases in children of older fathers [27,44]. In mouse models, whole-genome methylation analyses have similarly shown that sperm from older sires exhibit widespread DNA hypomethylation across numerous loci, including regions involved in neuronal differentiation. One study specifically reported the enrichment of REST/NRSF binding sites within hypomethylated regions [45]. Experimentally, this sperm DNA hypomethylation was associated with altered gene expression in embryos and abnormal behavioral outcomes in the offspring of old male mice, implying a causal epigenetic link [45].

Additionally, histone modifications and chromatin packaging in germ cells are affected by aging. During spermiogenesis, most histones are replaced by protamines, but a fraction of histones remain in specific genomic regions. Age-related disturbances in the histone-to-protamine transition have been noted. Paoli et al. found that aging human sperm had an increased protamine 1-to-protamine 2 ratio, indicative of anomalous protamination or retention of histones [31]. Aberrant protamine ratios can lead to less condensed sperm chromatin and higher susceptibility to DNA damage. Consistent with this, the sperm of older men shows higher rates of DNA fragmentation and oxidative DNA lesions [31,39]. Changes in specific histone marks in testicular cells with age have also been reported in rodent studies (e.g., altered H3K27 and H4K16 acetylation in aged spermatocytes, reflecting an aging chromatin state). One group previously demonstrated that histone modification patterns in spermatogenesis shift with age, paralleling the DNA methylation changes in sperm [42]. These epigenetic alterations could deregulate transcription in germ cells or affect the genomic imprinting and gene activation in the embryo post-fertilization.

In summary, male germline aging is accompanied by epigenetic reprogramming characterized by (1) changes in small RNAs (miRNA profiles shift, possibly affecting early developmental signaling; the piRNA pathway efficacy might decline), (2) accumulative DNA methylation changes (predominantly losses of methylation at certain loci in sperm DNA with advancing age [27]), and (3) altered chromatin packaging (protamine/histone imbalance and associated histone mark changes). These molecular alterations not only contribute to reduced fertility in the aging male but can also be transmitted as epigenetic information to the next generation, potentially impacting offspring health. Understanding these changes lays the foundation for developing biomarkers of testicular aging—such as specific sperm miRNA or DNA methylation signatures predictive of paternal age-related effects—and for designing interventions including lifestyle, pharmacological, or dietary strategies, to mitigate the adverse impact of aging on the male germline [46].

## 7. Translational and Clinical Implications

**Figure 2:**
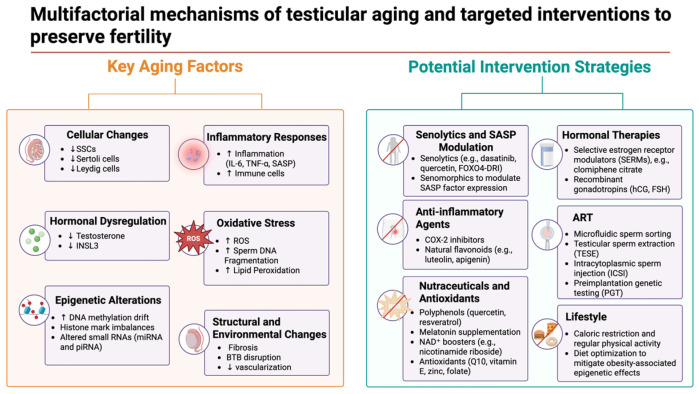
Multifactorial mechanisms of testicular aging and targeted interventions to preserve fertility. Created in BioRender. Kaltsas et al., 2025. Available online: https://BioRender.com/kyf94v7 (accessed on 1 June 2025). This schematic illustrates the key biological processes driving testicular aging and highlights evidence-based strategies to counteract them. Left panel: age-related alterations include the depletion of stem and somatic cells, hormonal decline (↓ testosterone and ↓ INSL3), epigenetic drift (DNA methylation, histone modifications, and small RNA changes), increased inflammation (↑ IL-6, TNF-α, and SASP factors), oxidative stress (↑ ROS and sperm DNA fragmentation), fibrosis, and disruption of the blood–testis barrier (BTB). Right panel: targeted interventions comprise senolytics (e.g., FOXO4-DRI), anti-inflammatory agents (COX-2 inhibitors and flavonoids), hormonal therapies (SERMs and gonadotropins), antioxidants and nutraceuticals (polyphenols, melatonin, and NAD⁺ boosters), assisted reproductive technologies (ICSI, TESE, and microfluidic sperm sorting), and lifestyle interventions (exercise and dietary optimization). Abbreviations: SSC = spermatogonial stem cell; SASP = senescence-associated secretory phenotype; BTB = blood–testis barrier; ROS = reactive oxygen species; NAD⁺ = nicotinamide adenine dinucleotide; ART = assisted reproductive technology.

The growing understanding of age-related molecular changes in the male germline and testicular microenvironment is driving new clinical and translational strategies. A key goal is to mitigate the decline in fertility and testicular function seen in older men by targeting the identified biological hallmarks—ranging from senescent cell accumulation and oxidative damage to hormonal and epigenetic alterations. Multiple approaches, both pharmacological and technological, are under investigation to bridge these bench discoveries to bedside interventions [47]. A visual summary of the key aging mechanisms and corresponding therapeutic strategies is presented in Figure 2.

### 7.1. Pharmacological and Nutraceutical Interventions

One promising avenue is the use of senolytic compounds to eliminate senescent cells that accumulate in the aging testis. By clearing these dysfunctional cells (and their pro-inflammatory secretions), the healthy niche needed for sperm production can be rejuvenated. In preclinical studies, treating aged mice with a senolytic cocktail of dasatinib plus quercetin (D + Q) dramatically reduced the burden of senescent cells in the testis and restored spermatogonial stem cell proliferation to youthful levels [33]. Notably, senescence was found mainly in the supportive somatic compartment (e.g., endothelial cells) rather than germ cells, and removing those senescent somatic cells rescued the support capacity for spermatogenesis [33]. This insight has sparked growing interest in senolytics and related senomorphics—which suppress the senescence-associated secretory phenotype—as potential therapies to rejuvenate the testicular microenvironment in aging males. Another novel example is a peptide senolytic (FOXO4-DRI), which was recently shown to alleviate testicular aging in mice by clearing senescent cells and improving testosterone levels [48], highlighting the potential of such targeted geroprotectors to treat late-onset hypogonadism. Complementing senolytics, general anti-aging drugs such as metformin (an AMPK activator and anti-inflammatory agent) or NAD+ boosters are also being considered to improve testicular cellular health, given evidence that metabolic interventions can impact the aging gonad. For instance, NAD+ levels decline with age in many tissues including testes; intriguing animal data show that experimentally inducing NAD+ deficiency in otherwise young mice impairs spermatogenesis, an effect fully reversed by niacin (vitamin B3) supplementation [49]. Similarly, the administration of nicotinamide riboside (NR) has been shown to alleviate testicular aging by restoring NAD+ levels, mitigating DNA damage in germ cells, and preserving normal spermatogenesis in a Qprt-deficient mouse model [50]. This suggests that maintaining metabolic fitness—e.g., through diet or supplements that sustain NAD+—might combat one driver of age-related male infertility.

Oxidative stress and chronic inflammation are other major targets of intervention, given their recognized roles in male reproductive aging [51]. Indeed, micronutrient–antioxidant therapies have been reported to boost sperm quality and in vitro fertilization (IVF) outcomes by neutralizing free radicals and protecting DNA integrity, further underscoring the therapeutic potential of antioxidant supplementation in ART cycles [52]. A variety of antioxidants, nutraceuticals, and anti-inflammatory compounds have shown protective effects on the aging testis in preclinical models. For example, long-term dietary polyphenols such as quercetin or resveratrol significantly reduced lipid peroxidation in aged rodent testes and boosted antioxidant enzyme activity [53]. These treatments also modulated key inflammatory pathways; aged Leydig cells in untreated animals showed elevated cyclooxygenase-2 (COX-2) expression coupled with reduced steroidogenic gene expression, whereas exposure to a COX-2 inhibitor or naturally occurring flavonoids (luteolin, apigenin) restored testosterone production capacity by suppressing COX-2–driven inflammation [54]. Likewise, the potent antioxidant hormone melatonin has been tested as a geroprotective therapy in animal models of testicular aging. Short-term melatonin administration in aged rats partially reverted testicular atrophy, increased the germinal epithelium thickness, and reduced apoptotic (TUNEL-positive) germ cells [55], consistent with melatonin’s anti-oxidative and anti-inflammatory actions. Another example is curcumin (a nutraceutical from turmeric), which in aged rats improved seminiferous tubule architecture and decreased germ cell apoptosis, although some studies noted these histological benefits did not always translate into higher serum testosterone [55]. While human data are still limited, these findings collectively underscore that antioxidant and anti-inflammatory therapies—ranging from diet-derived supplements to specialized drugs—may ameliorate the oxidative damage and inflammatory signaling that accumulate in the aging male reproductive tract. Some of these agents (e.g., co-enzyme Q10, vitamin E, zinc, and folate) are already empirically used as male fertility supplements in older or subfertile men, and clinical trials are beginning to evaluate their efficacy in improving semen quality and pregnancy outcomes [56]. Emerging “geroprotective” therapies, such as senolytics, are even now entering early human trials in other age-related conditions [57,58], raising the possibility that the next wave of clinical studies could test their utility in older men seeking to extend their fertile years.

### 7.2. Hormonal Modulation Strategies

Another translational approach focuses on the endocrine changes in the aging testis. With advancing age, men often experience a gradual decline in testosterone production due to a reduction in the number and function of Leydig cells [59,60,61]. However, conventional testosterone replacement therapy (TRT) is contraindicated in men who wish to maintain fertility because exogenous testosterone suppresses the pituitary gonadotropins required for spermatogenesis [60,61]. To navigate this, clinicians are repurposing hormonal modulators that can boost endogenous testosterone without impairing sperm output. A prime example is the off-label use of selective estrogen receptor modulators (SERMs) such as clomiphene citrate, which blocks estrogen feedback at the hypothalamus and thereby increases GnRH and gonadotropin release [62]. In men with age-related hypogonadism, clomiphene therapy elevates luteinizing hormone (LH) and follicle-stimulating hormone (FSH) levels, leading to increased intratesticular testosterone and stimulated sperm production [63]. SERM-based therapy has been effective in restoring physiological testosterone in older hypogonadal men while maintaining or enhancing sperm production [64,65], making it a valuable alternative to TRT for older men desiring paternity. In parallel, recombinant gonadotropins (hCG or FSH) are sometimes used to support spermatogenesis in men with borderline testicular function [66]; although not specific to aging, these can be considered on a case-by-case basis to overcome age-exacerbated gonadotropin insufficiency or to “kick-start” a sluggish spermatogenic process. Enhancing the testicular microvasculature and metabolism is another hormone-related angle: for instance, age-related testicular blood flow decline and tissue hypoxia might be addressed by lifestyle changes or medications that improve vascular health, indirectly benefiting hormone delivery and waste removal in the testis [62]. In parallel, emerging perspectives advocate for natural-product-derived therapies and antioxidant-based strategies to support testosterone production and manage male reproductive aging [67], particularly in cases where conventional TRT is contraindicated. Overall, hormonal and metabolic interventions informed by the biology of aging are being tailored to maintain the endocrine milieu needed for sperm production in older men, without the trade-offs that come with blanket hormone replacement.

### 7.3. Advanced Reproductive Technologies for Older Men

Concurrently, the assisted reproduction field is adapting techniques to address the unique challenges of advanced paternal age. Even with medical therapies, older men’s sperm often exhibit lower quality—including higher rates of DNA damage, oxidative lesions, and epigenetic aberrations—which can reduce fertilization capability and embryo development [27,68]. To counteract this, specialized sperm selection and assisted fertilization protocols have been developed for use in ART (assisted reproductive technology) contexts. One innovation is the use of microfluidic sperm-sorting devices to enrich sperm with better motility and genomic integrity. Since paternal aging is associated with increased sperm DNA fragmentation [68], microfluidic chips can filter out sperm carrying fragmented DNA by mimicking aspects of the natural selection in the female reproductive tract. Recent clinical evidence supports this approach: In IVF cycles using donor eggs, applying a microfluidic sperm selection (instead of standard density gradient processing) yielded significantly improved outcomes for older fathers. Notably, when fathers were ≥45 years old, microfluidic selection led to a higher embryo implantation rate (≈52% vs. 32%) and live birth rate (42% vs. 24%) compared to conventional sperm prep methods [68]. These findings have prompted the proposal that advanced paternal age be used as an indication for microfluidic sperm selection, to reduce sperm DNA damage in ART procedures [68]. Other sperm selection techniques are also being explored in this context: for example, magnetic-activated cell sorting (MACS) can remove apoptotic sperm (which often carry DNA breaks), and intracytoplasmic morphologically selected sperm injection (IMSI) uses ultra-high magnification to select sperm with the best nuclear morphology, which may correlate with genomic integrity [69]. While more studies are needed, such tools could increase the odds of healthy pregnancy with older sperm by ensuring that the sperm injected or inseminated are the “top tier” of what an older male ejaculate has to offer.

In addition to sperm selection, modifications in fertilization techniques themselves are used to accommodate older fathers. Intracytoplasmic sperm injection (ICSI) has become the preferred fertilization method for couples with an older male partner since ICSI can bypass potential age-related declines in sperm’s ability to penetrate the egg. In fact, some clinics recommend ICSI by default for men over ~50 years, backed by data showing higher fertilization rates in older men with ICSI compared to conventional IVF [4]. ICSI also allows the use of testicular sperm extraction in cases where ejaculated sperm quality is very poor; retrieving sperm directly from the testis of an older man can sometimes yield cells with less DNA fragmentation than in the ejaculate [70]. Moreover, the awareness of epigenetic and genetic risks associated with older sperm has led to the increased use of preimplantation genetic testing (PGT) on embryos fertilized by older men with the aim of screening out aneuploid embryos or those with de novo mutations [4]. Taken together, these refinements in ART—from enhanced sperm selection to tailored fertilization and genetic screening protocols—represent direct translational responses to the challenges uncovered by molecular studies of aging sperm.

### 7.4. Linking Omics Findings to Translational Strategies

Crucially, each of the above interventions is grounded in specific molecular or transcriptomic insights about aging in the male reproductive system. For example, the transcriptomic profiling of aging testes has revealed the upregulation of inflammatory cytokines and stress-response genes in somatic support cells, which in turn has inspired trials of anti-inflammatory drugs (such as COX-2 inhibitors and nutraceuticals) to alleviate this inflammation and restore normal gene expression [48,54]. Likewise, the discovery of epigenetic “drift” in older sperm—such as age-associated DNA methylation changes and altered histone retention [39]—has raised awareness of the potential impact of paternal aging on offspring via epigenetic mechanisms. While no pill can yet reverse an older man’s sperm methylation age, this knowledge is prompting recommendations for older prospective fathers to optimize other factors (diet, exercise, and avoiding tobacco and toxic exposure) to minimize additional epigenetic insults to their germ cells [39]. It has also motivated exploratory research into whether interventions such as folate supplementation or HDAC/Sirtuin-activating compounds could stabilize the sperm epigenome in aging. Another clear bench-to-bedside connection is the role of inflammatory signaling and the testicular immune environment: Studies show that aging testes develop a mild chronic inflammatory state (sometimes termed “inflammaging”), with elevated interleukins and macrophage activity that can impair spermatogenesis [51,54]. This has led to trials of anti-inflammatory agents (e.g., low-dose NSAIDs and natural anti-inflammatories) and even the consideration of testicular anti-senescent therapies (such as local senolytic application) to break the cycle of inflammation and tissue damage. In sum, basic scientific findings—from single-cell RNA-seq maps of the aging testis to proteomic analyses of aging semen—are continually informing the development of targeted interventions [71]. These efforts ensure that translational research is not happening in a vacuum but rather is directly guided by the mechanistic causes of age-related fertility decline identified in the lab.

## 8. Future Research Directions

Several key questions remain unanswered, warranting further investigation into the male germline and testicular microenvironment with advancing age. Addressing these gaps will not only deepen our fundamental understanding but also guide translational strategies to maintain male reproductive health.

### 8.1. Reversibility of Epigenetic Alterations

One critical question is whether age-associated epigenetic changes in the male germline are reversible or permanent. Advanced paternal age is known to correlate with altered sperm DNA methylation patterns, histone modifications, and small non-coding RNA profiles, changes that have been hypothesized to be “dynamic over years, yet stable over days and months, and likely irreversible” [39]. However, it remains to be determined whether interventions can restore a more “youthful” epigenetic state to aging sperm. Emerging evidence in other contexts suggests epigenetic plasticity—for example, paternal exercise can modulate DNA methylation in germ cells, with sperm from exercised males showing specific methylation changes that are transmitted to offspring [72]. Future studies should examine whether lifestyle modifications or pharmacological agents (such as epigenetic drugs or dietary supplements) could similarly reset or slow the epigenetic clock in aging male germ cells. Establishing the reversibility (or irreversibility) of these epigenetic alterations is crucial as it would open the door to interventions aimed at rejuvenating the sperm epigenome and improving fertility and offspring outcomes in older fathers.

### 8.2. Causal vs. Correlative Changes in Testicular Aging

Another major challenge is disentangling which age-related changes in the testes are causal drivers of functional decline versus those that are merely correlative byproducts of aging. The aging testis exhibits a host of molecular and cellular alterations—for instance, pro-inflammatory cytokine levels rise, and ROS accumulate in the aging gonad [53]—but the actual impact of these changes on fertility and germ cell viability remains incompletely understood [53]. As one review notes, it is inherently difficult to determine which molecular or morphological characteristics represent mechanisms of accelerated testicular aging and which should be regarded simply as markers of biological age [53]. Going forward, research must focus on establishing causation, for example, using targeted interventions (such as reducing oxidative stress or inflammatory signaling in animal models) to assess whether this attenuates age-related sperm defects. Similarly, longitudinal studies in humans and model organisms could help identify early molecular predictors of testicular aging that truly drive the decline. By distinguishing cause from correlation, we can prioritize the key pathways (e.g., DNA damage accumulation, hormonal changes, or immune cell infiltration) that need to be targeted to prevent age-induced testicular dysfunction.

### 8.3. Interventions to Prevent or Delay Sperm Aging

There is a pressing need to explore medical, lifestyle, and dietary interventions that might slow down or prevent the aging of the male germline. Healthy lifestyle choices are a promising starting point: Caloric restriction and exercise are known to delay systemic aging [73], and specifically, paternal diet and fitness before conception can influence sperm epigenetic makeup and offspring health [74]. For example, paternal obesity and poor diet are associated with detrimental epigenetic changes in sperm linked to metabolic disorders in offspring [74], implying that improving diet, exercise, and weight in older prospective fathers could conversely benefit sperm quality and reduce age-related risk factors. Besides lifestyle, various pharmacological or nutraceutical approaches have been proposed to counteract testicular aging. Antioxidant and anti-inflammatory treatments are of particular interest, given the role of oxidative damage and inflammation in aged testes [53]. Indeed, recent work highlights the potential of interventions ranging from non-steroidal anti-inflammatory drugs to nutraceuticals (e.g., vitamins, polyphenols, and probiotics) that exhibit anti-oxidative and anti-apoptotic properties, some of which are already being investigated to delay or prevent testicular aging [53]. Rigorous in vivo studies are needed to test which supplements or drugs can preserve sperm count, quality, or DNA integrity in aging males and to establish safe guidelines for their use. It is likely that no single therapy will suffice; combination approaches may yield the best outcomes, for example, pairing lifestyle modification with targeted pharmacotherapy. More research in animal models is essential to identifying effective intervention combinations and dosing strategies, which can then be translated into clinical trials. Given the limited availability of human testicular biopsies from healthy aged men, innovative and translational research designs (such as noninvasive biomarker studies or opportunistic trials in older IVF patients) will be critical [53]. Overall, developing evidence-based interventions to forestall germline aging could have profound benefits for extending male fertility and ensuring healthier offspring.

### 8.4. Preserving and Restoring SSC and Niche Function

Strategies to preserve or restore the function of SSCs and the aging testicular niche represent another key avenue for future research. Age-related infertility in males is thought to stem in part from a decline in the supportive microenvironment (niche) that nurtures SSCs. Notably, experiments in mice have shown that the intrinsic potential of SSCs may not be the limiting factor—old SSCs transplanted into a young testicular environment can continue to sustain spermatogenesis far beyond the normal lifespan of the donor [75]. This finding implicates niche deterioration (e.g., the loss of critical growth factors, accumulation of senescent somatic cells, or fibrosis of the seminiferous tubules) as a primary driver of germline aging. Future work should aim to identify the specific changes in the aging niche that compromise SSC self-renewal and differentiation. For instance, declines in factors such as GDNF or FGF2 (which are vital for SSC maintenance) or increases in inhibitors (such as inflammatory cytokines) could be targeted. Therapeutic approaches to rejuvenating the niche are an exciting prospect and might include delivering pro-stem-cell factors to the aged testis, eliminating senescent or inhibitory cells (e.g., via senolytic drugs), or bioengineering scaffolds to support stem cell function. In parallel, advances in SSC-based therapies offer potential routes to restoring fertility. Techniques such as SSC transplantation, testicular tissue grafting, and even in vitro spermatogenesis have all achieved important milestones in preclinical models [76]. To date, these methods remain experimental and have not been applied clinically for age-related infertility, but they illustrate what may be possible. Moving forward, it will be important to translate these approaches into translational research and eventually clinical practice—for example, by developing protocols for autologous SSC transplantation in older men or agents that can safely reactivate dormant stem cells in situ. Ultimately, protecting the spermatogonial stem cell pool and maintaining a healthy niche could prolong the reproductive lifespan of males or even restore fertility in those experiencing age-related decline.

### 8.5. Transgenerational Implications of Male Germline Aging

Finally, a deeper understanding is needed of the transgenerational impacts of male germline aging and the mechanisms mediating these effects. It has become clear that paternal age not only affects the father’s fertility but also the health and development of the next generation. Advanced paternal age has been associated with subtle increases in risk for neurodevelopmental disorders and other health issues in offspring, potentially via epigenetic alterations in sperm that escape normal reprogramming in the early embryo [39]. Indeed, there is growing evidence that an aging father’s sperm carries epigenetic memory—in the form of DNA methylation changes, retained histone marks, or altered small non-coding RNA (sncRNA) payloads—which may perturb gene regulation in the embryo and affect offspring phenotype [39]. A key unresolved question is the extent to which these epigenetic aberrations contribute to observed effects in offspring and whether such changes persist across subsequent generations, indicating true transgenerational inheritance. Studies of environmental exposure provide a framework for this; for example, paternal exposure to endocrine-disrupting chemicals has been linked to the transgenerational transmission of increased disease susceptibility and reproductive disorders via epigenetic changes in the germline [74]. Similarly, chronic stress in male mice can induce sperm DNA methylation changes that evade embryonic erasure and lead to metabolic and behavioral effects in offspring and even grand-offspring [77]. These models underscore that the sperm epigenome can carry information about the father’s condition (age, stress, diet, etc.) forward to progeny. Regarding male aging, we need focused research to identify which age-related molecular signatures in sperm are passed to children and grandchildren, and through what carriers (e.g., particular methylation marks, non-coding RNAs, or residual proteins). Elucidating these mechanisms will require integrative approaches—the genome-wide epigenomic profiling of sperm from aging males, CRISPR-based editing or epigenetic editing to test specific marks, and carefully controlled animal breeding studies—to observe multigenerational outcomes. This knowledge is not only of academic interest but also of practical significance. If certain harmful epigenetic alterations are shown to be heritable, targeted interventions—such as preconception treatments for older fathers or embryo screening for epigenetic abnormalities—could be developed to mitigate their impact. Clarifying the transgenerational risks associated with male germline aging will enhance medical counseling, including guidance on the optimal timing of parenthood and preconception health, and may ultimately support strategies to safeguard the well-being of future generations.

## 9. Conclusions

The gathered evidence demonstrates that advancing paternal age elicits multifaceted alterations in the male germline and testicular milieu, encompassing epigenetic reprogramming, declining spermatogonial stem cell populations, and reconfigured intercellular signaling networks. Gene expression across spermatogenesis steadily shifts, with some older testes retaining undifferentiated spermatogonia that fail to mature into functional sperm. Sertoli cells undergo both numeric decline and a pronounced shift toward a pro-senescent, inflammatory phenotype, while Leydig cells experience attenuated steroidogenic function, ultimately impairing testosterone and INSL3 production. These changes are further compounded by fibrosis, disrupted barrier integrity, and expanded immune cell infiltration in the aging testis, thereby weakening the supportive niche required for continuous fertility. Moreover, notable disruptions in small RNA profiles, DNA methylation patterns, and chromatin packaging in sperm suggest that epigenetic drift contributes not only to deteriorating sperm quality but also to potential transgenerational consequences impacting offspring development.

Collectively, these findings delineate the gradual but pervasive nature of male reproductive aging, which contrasts sharply with the abrupt ovarian failure characteristic of female menopause. Recent multi-omics approaches have illuminated the intricate interactions between germ cells, Sertoli cells, Leydig cells, and the broader testicular environment, facilitating deeper insights into the root causes of reduced fertility, heightened miscarriage risks, and developmental perturbations observed in children of older fathers. Targeting senescence pathways, bolstering essential niche signals, and managing inflammation may offer plausible avenues to mitigating or delaying these adverse outcomes. As reproductive aging increasingly intersects with societal trends of delayed fatherhood, further investigation of these molecular underpinnings remains crucial for devising effective strategies that preserve fertility and safeguard offspring health.

## Figures and Tables

**Table 1:** Summary of key primary studies on testicular aging, ordered by species.

Study (First Author, Year)	Population (Species, Sample Size)	Age Groups/Time Points	Methodology	Sample/Cell Type	Main Findings
Cui et al., 2025 [25]	Humans (*n* = 35)	21–69 y	scRNA-seq	Whole testis	Two aging waves: fibrosis (30 s), metabolic dysregulation (50 s) Somatic > germ cell aging
He 2024 [26]	Humans (*n* = 23)	Young: <60 y (*n* = 14) Old: ≥60 y (*n* = 9)	scRNA-seq + CellCall + SASP gene profiling	Leydig cells	↑IGFBP7 in aged Leydig cells ↓ germ–Leydig signaling, ↑senescence
Bernhardt 2023 [27]	Humans (*n* = 73)	25.8–50.4 y	RRBS + validation via pyrosequencing	Sperm	1565 DMRs (74% hypo) Linked to infertility and neurodevelopment
De Sena Brandine et al., 2023 [28]	Humans (*n* = 10)	Samples 10–18 years apart	WGBS + mixed-effects modeling	Sperm	Global sperm hypomethylation Promoter HMRs expand with age
Nie et al., 2022 [29]	Humans (*n* = 12)	17–22 y vs. 62–76 y	Single-cell RNA-seq + CellChat + validation assays	Whole testis	Somatic cell dysregulation (inflammation, metabolism) SSCs stable; BMI ↑ effects
Alfano et al., 2021 [30]	Human testis from infertile men with idiopathic GCA (*n* = 3 iNOA) vs. fertile controls	32–41 y vs. controls	scRNA-seq + histology + hormone/immune profiling	Somatic cells from whole testis tissue (Sertoli, Leydig, peritubular myoid, endothelial, stromal, immune)	Sertoli and Leydig immaturity, fibrosis, senescence ↑DLK1 ↓INSL3 Niche aging
Paoli 2019 [31]	Human males (*n* = 2626 for semen analysis; *n* = 40 vs. 40 for molecular)	20–40 y vs. 50–81 y	Semen analysis, TUNEL, RT-qPCR (PRM1/2), miRNA profiling	Ejaculated sperm and seminal plasma	↑DNA fragmentation, ↓PRM1/2, miR-122/371/146a Chromatin instability
Liu et al., 2025 [32]	Mice (C57BL/6; *n* = 3 young vs. 3 old for scRNA-seq; *n* = 10 for BHB studies)	2 mo vs. 24 mo	scRNA-seq, qPCR, AAV-Cre/overexpression, BHB supplementation	Leydig cells	↓Hmgcs2 in aged Leydig → ↓testosterone, ↑p21 BHB reversed aging signs
Kawahara et al., 2025 [24]	Mice (C57BL/6J; *n* = 3 per age group; *n* = 800–1000 tubule sections)	3–4 mo vs. 15–20 mo vs. 25–28 mo	scRNA-seq, lineage tracing, intravital imaging	Undifferentiated spermatogonia (GFRα1⁺ SSCs)	Clonal SSCs expand with age, fail to form sperm ↓Egr4, ↓Cops5 expression in aged SSCs
Ozawa 2023 [33]	Mice (C57BL/6J; *n* = 8–13 per group)	2 mo vs. >24 mo	Histology, IHC, SA-β-gal, RNA-seq, GSC-EC co-culture	Testicular endothelial cells (ECs)	Senescent ECs lose GSC support ↑SASP D + Q rejuvenates function
Kanatsu-Shinohara et al., 2019 [34]	Mouse SSC cultures (5M vs. 60M; *n* = 3–12); aged mouse and rat testes	5 mo vs. 60 mo (in vitro); 2 y in vivo	Long-term SSC culture, transplantation, RNA-seq, ChIP-seq, metabolic assays	Cultured SSCS and SSCs from aged mice/rats	Long-term SSCs: hyperproliferative, non-functional ↑JNK, ↓PRC2 Glycolytic shift
Fice & Robaire 2023 [21]	Brown Norway rats (*n* = 5/group; RS purity ≥88%)	5.3 mo vs. 19.2 mo	RNA-seq (RS); GO, IPA, PCA	Round spermatids	220 DEGs in round spermatids (211 ↑, 9 ↓) Altered motility, ROS, epigenetic marks, and Sertoli–germ signaling
Luo 2023 [35]	Mice (C57BL/6; *n* = 6/group; + p38LCKO line)	6 mo vs. 18 mo; + HFD mice for 24 wks	ELISA, qPCR, WB, IF, IHC, SA-β-Gal, scRNA-seq (human)	Leydig cells	p38 MAPK drives Leydig aging Knockout → ↑Star/Cyp11a1/testosterone, ↓p21
Huang et al., 2023 [23]	Cynomolgus monkeys (*n* = 4 young, *n* = 4 old)	5–6 y vs. 18–21 y	snRNA-seq (70,400 nuclei); IF, IHC, SA-β-Gal, transcriptome-wide analysis	SSCs, Sertoli cells, Leydig cells	SSCs deplete Sertoli cells show most DEGs, ↑noise ↓WT1/ZO-1; WT1 KD → senescence

Abbreviations: scRNA-seq: single-cell RNA sequencing; snRNA-seq: single-nucleus RNA sequencing; WGBS: whole-genome bisulfite sequencing; RRBS: reduced representation bisulfite sequencing; DMR: differentially methylated region; SSC: spermatogonial stem cell; LC: Leydig cell; TPC: testicular peritubular cell; GCA: germ cell aplasia; PRM1/2: protamine 1/protamine 2; HMR: hypomethylated region; SA-β-gal: senescence-associated β-galactosidase; BHB: β-hydroxybutyrate; JNK: c-Jun N-terminal kinase; PRC2: Polycomb repressive complex 2; DEG: differentially expressed gene; ROS: reactive oxygen species; GO: Gene Ontology; IPA: Ingenuity Pathway Analysis; HFD: high-fat diet; IF/IHC: immunofluorescence/immunohistochemistry; WB: Western blot; TSS: transcription start site; AR: androgen receptor; NRG1: neuregulin 1; DLK1/INSL3: delta-like 1 homolog/insulin-like 3; ZO-1: zonula occludens-1; y: years; mo: months; wks: weeks; ↑: increased/upregulated; ↓: decreased/downregulated; →: leads to/causes/is associated with.

## Data Availability

No new data were created or analyzed in this study.
